# TGN1412 Induces Lymphopenia and Human Cytokine Release in a Humanized Mouse Model

**DOI:** 10.1371/journal.pone.0149093

**Published:** 2016-03-09

**Authors:** Sabrina Weißmüller, Stefanie Kronhart, Dorothea Kreuz, Barbara Schnierle, Ulrich Kalinke, Jörg Kirberg, Kay-Martin Hanschmann, Zoe Waibler

**Affiliations:** 1 Junior Research Group „Novel Vaccination Strategies and Early Immune Responses”, Paul-Ehrlich-Institut, Langen, Germany; 2 Division of Immunology, Paul-Ehrlich-Institut, Langen, Germany; 3 Division of Virology, Paul-Ehrlich-Institut, Langen, Germany; 4 Institute of Experimental Infection Research, TWINCORE, Centre of Experimental and Clinical Infection Research—a joint venture between the Hannover Medical School (MHH) and the Helmholtz Centre for Infection Research (HZI), Hannover, Germany; 5 Division of Biostatistics, Paul-Ehrlich-Institut, Langen, Germany; University of California, San Francisco, UNITED STATES

## Abstract

Therapeutic monoclonal antibodies (mAbs) such as the superagonistic, CD28-specific antibody TGN1412, or OKT3, an anti-CD3 mAb, can cause severe adverse events including cytokine release syndrome. A predictive model for mAb-mediated adverse effects, for which no previous knowledge on severe adverse events to be expected or on molecular mechanisms underlying is prerequisite, is not available yet. We used a humanized mouse model of human peripheral blood mononuclear cell-reconstituted NOD-RAG1^-/-^Aβ^-/-^HLADQ^(tg+ or tg-)^IL-2Rγc^-/-^ mice to evaluate its predictive value for preclinical testing of mAbs. 2–6 hours after TGN1412 treatment, mice showed a loss of human CD45^+^ cells from the peripheral blood and loss of only human T cells after OKT3 injection, reminiscent of effects observed in mAb-treated humans. Moreover, upon OKT3 injection we detected selective CD3 downmodulation on T cells, a typical effect of OKT3. Importantly, we detected release of human cytokines in humanized mice upon both OKT3 and TGN1412 application. Finally, humanized mice showed severe signs of illness, a rapid drop of body temperature, and succumbed to antibody application 2–6 hours after administration. Hence, the humanized mouse model used here reproduces several effects and adverse events induced in humans upon application of the therapeutic mAbs OKT3 and TGN1412.

## Introduction

Therapeutic monoclonal antibodies (mAbs) are approved for many clinical indications including cancer, immunological disorders, transplant rejection, and infectious diseases. Currently, there are 26 mAbs marketed in Europe and 27 mAbs marketed in the US and it is estimated that ~350 mAbs are in the pipeline being evaluated in clinical studies [1]. Nevertheless, although mAbs are potent and target-specific reagents, they may cause severe adverse effects when administered in vivo.

TGN1412, a superagonistic, humanized, CD28-specific IgG4 was applied in March 2006 during a first-in-human clinical trial to 6 healthy volunteers. Briefly after administration, all 6 volunteers experienced severe adverse effects such as fever, headache, hypotension, and lymphopenia, and ultimately all suffered from a multi-organ-failure. These severe adverse events could be attributed to the induction of a cytokine release syndrome (CRS), a life-threatening systemic release of cytokines [2]. Another mAb for which the induction of CRS has been reported, particularly upon first-dose administration, is muromonab-CD3 (Orthoclone OKT3^®^), a murine IgG2a used to treat acute organ rejection [3]. OKT3 is directed to the human T cell receptor-CD3 complex on the surface of circulating T cells. Meanwhile, manufacturing of this antibody was discontinued since other treatment options with comparable efficacy but fewer side effects became available [1].

The disastrous outcome of the first-in-human clinical trial of TGN1412 put the predictive value of preclinical animal models into question and there is an ongoing debate on whether or not the severe adverse events induced were predictable by the preclinical studies conducted [4, 5]. Studies in rodents initially indicated that application of CD28-specific superagonistic mAbs can ameliorate autoimmune and inflammatory diseases ([6, 7] and reviewed in [8]). Using JJ316 (a homolog to TGN1412; a mouse IgG1 mAb directed to rat CD28), beneficial effects of the treatment on EAE disease outcome was associated with expansion of CD4^+^ regulatory T cells and release of anti-inflammatory cytokines such as interleukin (IL)-10 [6, 9].

Safety and toxicology studies for TGN1412 were conducted in rhesus and cynomolgus monkeys. Even though monkeys received a dose of TGN1412 which was up to 500-fold higher as applied in the first-in-human clinical trial, “no TGN1412-related signs of toxicity, hypersensitivity or systemic immune system deviation were observed in these studies” [10]. In contrast to the first-in-human clinical trial [2], in none of the animal models employed before the trial, lymphopenia was observed upon TGN1412 injection [10]. Moreover, upon single dose application of TGN1412 to monkeys, no cytokine release has been reported. Upon repeated dose treatment, a moderate increase in serum IL-2, IL-5, and IL-6 was observed in individual animals but no induction of tumor necrosis factor (TNF)-α and interferon (IFN)-γ [10], two cytokines which are most indicative for a CRS [11].

Taken together, the TGN1412 incidence indicated that preclinical models investigated were not necessarily predictive for severe adverse events such as lymphopenia and CRS. Much effort was spent to identify in vitro settings enabling TGN1412-mediated T cell activation. However, molecular mechanism could only be identified retrospectively with the knowledge of adverse effects mediated by the mAb. A predictive model for mAb-mediated adverse effects, for which no previous knowledge on molecular mechanisms involved is prerequisite, is not available yet. Here, we evaluated the predictive value of humanized mice for preclinical testing of mAbs. Investigating TGN1412 and OKT3 in humanized mice we recapitulate key effects that were observed upon application of the mAbs in humans, such as the induction of lymphopenia and the induction of human cytokine release.

## Materials and Methods

### Ethics statement

Mouse experimental work was carried out in strict compliance with regulations of German animal welfare. The protocol was approved by the Regierungspräsidium Darmstadt (permit number: F107/86). Blood was withdrawn under anesthesia, and all efforts were made to minimize suffering. Ethical approval for research involving human cells was not necessary since buffy coats are commercially available from the Deutsche Blutspendedienst.

### Mice

NOD.Cg-Rag1^tm1Mom^ Il2rg^tm1Wjl^ H2-Ab1^tm1Doi^ mice, either being non-transgenic or carrying the TgN(HLA-DQA1, HLA-DQB1)1Dv allele, (NRG) were established from NOD.Cg-Rag1^tm1Mom^ Il2rg^tm1Wjl^/SzJ and NOD.Cg-Prkdc^scid^ H2-Ab1^tm1Doi^ TgN(HLA-DQA1, HLA-DQB1)1Dv/SzJ breeders. They were bred under SPF conditions at the Zentrale Tierhaltung of the Paul-Ehrlich-Institut. Mouse experimental work was carried out using 8 to 12 weeks old mice in compliance with regulations of German animal welfare.

### PBMC purification and mice reconstitution

**Fig 1:**
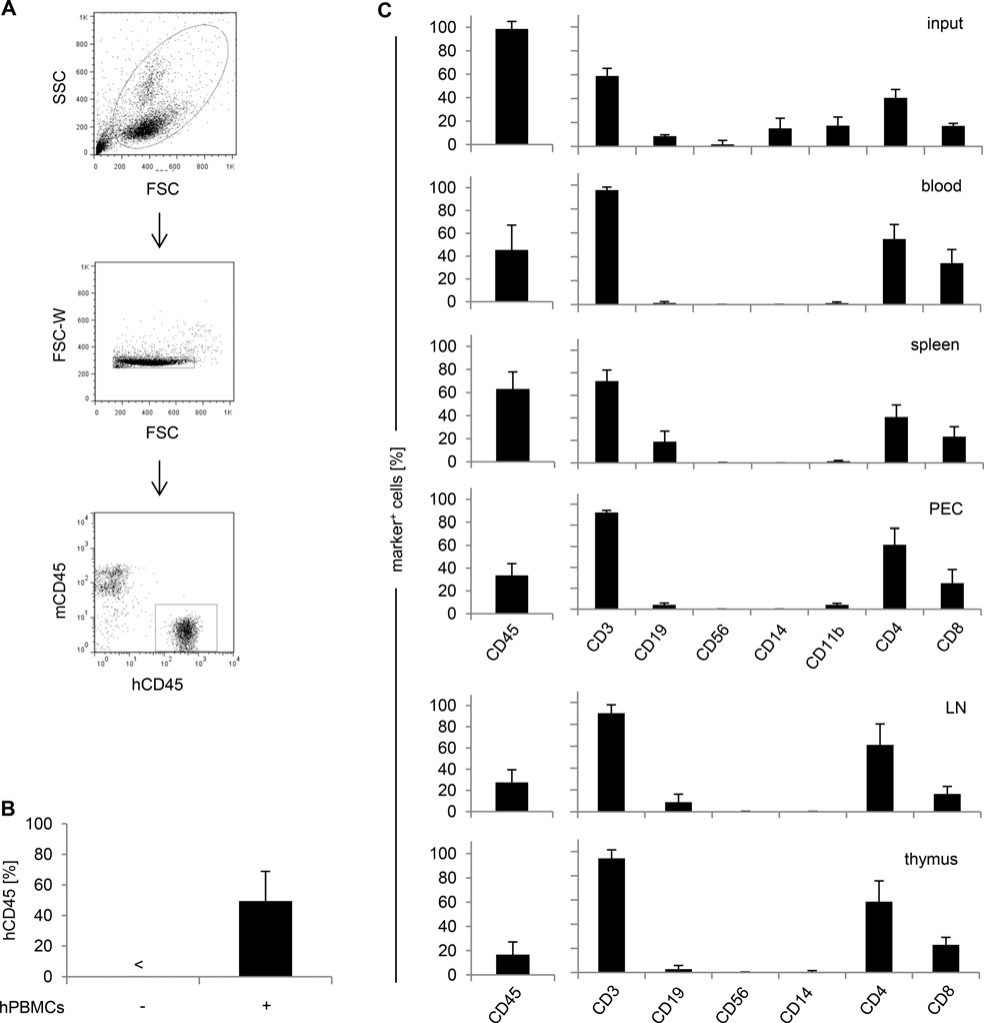
Human T cells are the predominant cell population within the peripheral blood in humanized mice. NRG mice were injected i.v. with 6.5x10^7^-1x10^8^ hPBMCs. **(A)** All flow cytometric analyses throughout this study were performed by gating on living cells in the FSC/SSC plot, excluding cell doublets in an FCS/FSC-W plot, and gating on hCD45^+^ cells. **(B)** Percentages of hCD45^+^ cells in the peripheral blood of NRG mice were analyzed by flow cytometric analysis before reconstitution (n = 3) or on day 8–16 after reconstitution (n = 73). <; not detectable. **(C)** Cell composition within hCD45^+^ cells of freshly isolated hPBMCs (input; n = 6) or peripheral blood (n = 54), spleen (n = 14), PEC (n = 6), LN (n = 9), and thymus (n = 9) of humanized mice on day 8–16 after repopulation was analyzed by flow cytometric analysis. Data shown in (A) are representative for all flow cytometric analyses performed throughout this study. Data shown in (B) are taken from 1–13 independent experiments. Data shown in (C) are taken from 2–7 independent experiments.

Human peripheral blood mononuclear cells (hPBMCs) were isolated from buffy coats from healthy donors (Blutspendedienst Frankfurt am Main, Germany) by Ficoll (Biochrom, Berlin, Germany) density gradient centrifugation. After washing twice with PBS (Biochrom, Berlin, Germany) cell suspensions of 200 μl containing 6.5x10^7^-1x10^8^ hPBMCs were injected intravenously (i.v.) in NRG recipients. Following hPBMC injection, mice were observed daily for weight loss, general appearance of the fur, and mobility. Mice were considered reconstituted and are referred to as “humanized” when hCD45^+^ in the peripheral blood exceeded 15%. This was usually between day 8 and 16 after reconstitution with varying reconstitution efficacies (compare Fig 1B). At that time point, mice were injected with either the monoclonal antibodies (mAb) or PBS immediately. Before and 2–6 hours after mAb treatment blood was collected for cytokine detection. This reflects the time point mice had to be sacrificed due to severe signs of illness. All mice were used before the onset of severe GvHD. Within one experiment, treatment- and control-groups were reconstituted with PBMCs from the same donor. For all experiments both DQ transgenic and DQ non-transgenic NOD.Cg-Rag1^tm1Mom^ Il2rg^tm1Wjl^ H2-Ab1^tm1Doi^ mice were used. No differences in reconstitution efficacy and in all assays where detectable when DQ transgenic and DQ non-transgenic mice were compared (data not shown).

### Antibody application

Usage of the superagonistic anti-CD28 mAb TGN1412 was kindly permitted by TheraMAB GmbH (Würzburg, Germany). Murine anti-CD3 mAb Orthoclone OKT3 was purchased from Janssen-Cilag (Neuss, Germany). mAb Herceptin was purchased from Roche. Chimeric anti-human TNF-α mAb Remicade was purchased from Janssen Biotech (USA). Humanized mice were injected i.v. with 1 μg TGN1412, 20 μg TGN1412, or 20 μg OKT3 per 10 grams body weight in a maximal volume of 200 μl. As negative control mice were injected i.v. with 200 μl PBS or 20 μg Herceptin per 10 gram body weight in a maximal volume of 200 μl.

For calculating the human equivalent dose of an reagent for usage in mice, conversion can be based on either body surface area or body weight [12]. The dosages of TGN1412 and OKT3 applied to humanized mice were calculated according the FDA guideline for calculation of the human equivalent dose based on body surface area. Briefly, TGN1412 was applied at 0.1 mg/kg in the first-in-human clinical trial [2]. Using 20 μg TGN1412 per 10 grams body weight in mice corresponds to 1.6-fold this dosage. OKT3 was applied at 5 mg/60 kg in patients [13]. Using 20 μg OKT3 per 10 grams body weight in mice corresponds to 1.95-fold this dosage. Of note, neither TGN1412 nor OKT3 bind to murine CD45^+^ cells (data not shown and [14]). For blocking human TNF-α, mice were treated i.v. with 10 μg anti-human TNF-α mAb Remicade. This dosage neutralizes 7.5 ng/ml TNF-α [15] and is hence sufficient to prevent cross-reactivity of the human cytokine with the murine system (OKT3 or TGN1412 treatment induces up to 0.67 ng/ml human TNF-α release in humanized mice). 1 hour after anti-human TNF-α mAb pre-treatment humanized mice were injected with OKT3 or TGN1412 as described above.

### Cell isolation

Splenocytes were harvested by dissociation of the spleen into single-cell suspensions by squeezing with a pipette after opened the splenic capsule. Cells were filtered through a 70 μm sieve (Becton Dickinson, Heidelberg, Germany) and centrifuged at 1200 rpm for 5 minutes. Red blood cells were lysed with red blood cell lysing buffer (Sigma, Missouri, USA). PEC (peritoneal exudate cells) were collected following injection of 5 ml PBS into the peritoneal cavity. Single-cell suspensions were prepared from LN (lymph nodes) and thymus by mashing through a 70 μM sieve.

### Flow cytometric analysis

The phenotype of human lymphocytes recovered from peripheral blood, spleen, PEC, LN, or thymus of reconstituted mice was analyzed by flow cytometry. For staining, cells were incubated first with mouse Fc block (clone 2.4G2, BD Pharmingen, Heidelberg, Germany) for 20 min at room temperature to exclude unspecific binding of mouse anti-human FACS-antibodies to mouse Fc receptors. Cells were stained for 20 min at 4°C with anti-human FACS-antibodies targeting the following cell-surface markers: anti-CD3-APC (clone UCHT1), anti-CD4-APC (clone RPA-T4), anti-CD4-APC-Cy7 (clone RPA-T4), anti-CD8-PE-Cy7 (clone RPA-T8), anti-CD14-Pacific blue (clone M5E2), anti-CD19-PE-Cy5 (clone HIB19), anti-CD25-PE (clone M-A251, data not shown), anti-CD45-PE (clone HI3), anti-CD45RA-FITC (clone HI100, data not shown), anti-CD45RO-APC (clone UCHL1, data not shown), anti-CD56-PE-Cy5 (clone B159), anti-CD69-FITC (clone FN50, data not shown; all from BD Pharmingen, Heidelberg, Germany), anti-CD11b-FITC (clone M1/70.15.11.5; Miltenyi Biotec, Bergisch Gladbach, Germany), anti-CD38-PE (clone HIT2, data not shown), anti-CD44-FITC (clone BJ18, data not shown), and anti-CD62L-APC-Cy7 (clone DREG-56, data not shown; all from BioLegend, San Diego, USA). After staining peripheral blood lymphocytes, FACS lysing solution (BD Pharmingen, Heidelberg, Germany) was used to lysate red blood cells. Cells were washed and analyzed using LSRII flow cytometer (Becton Dickinson, Heidelberg, Germany) and the BD FACS Diva software (BD Biosciences, Heidelberg, Germany) or FlowJo software (Tree Star, Ashland, USA). Freshly isolated hPBMCs obtained directly after density gradient centrifugation were used as control. Peripheral blood cells from non-reconstituted mice were used as negative controls. Rat anti-mouse CD45-FITC (clone 30-F11, BD Pharmingen, Heidelberg, Germany) was used for detection of leucocytes of murine origin.

To determine absolute cell counts (data not shown), 15 μl blood was mixed with 15 μl counting beads (approximately 1000 beads/μl; Invitrogen, Karlsruhe, Germany). After treatment with FACS lysing solution (BD Biosciences, Heidelberg, Germany), samples were subjected to FACS analysis with the acquisition of 5000 counting beads. Therefore, data correspond to approximately 5 μl blood.

For analyzing CD3 expression on human T cells after mAb application to reconstituted mice, cells were fixed with 1% PFA (Merck, Darmstadt, Germany) to prevent internalization of CD3 after OKT3-stimulation, 1 μg/ml OKT3 was exogenously added or cells were left untreated, and incubated for 20 min at room temperature. After washing, cells were stained with 2 μg/ml anti-mouse IgG mAb (Dianova, Hamburg, Germany) for 20 min at 4°C. After washing, cells were finally stained with anti-CD45, anti-CD4, and anti-CD8 FACS-antibodies as described above. Controls verified that fixation prior to staining did not change the staining (data not shown). The receptor occupancy of CD3 or CD28 by OKT3 or TGN1412, respectively, was analyzed by exogenously adding OKT3 or TGN1412 to cells isolated from spleen, PEC, LN, and thymus, and analyzed as described above. For the detection of OKT3- or TGN1412-binding to their target CD3 or CD28 in blood, spleen, PEC, LN, and thymus, cells were stained with 2 μg/ml anti-mouse IgG mAb or anti-human IgG mAb (both from Dianova, Hamburg, Germany) for 20 min at 4°C, respectively. After washing, cells were finally stained with anti-CD45, anti-CD4, and anti-CD8 FACS-antibodies as described above.

### Quantification of human and murine cytokine production

Human cytokines in mice sera were analyzed by human FlowCytomix Th1/Th2 11plex kit (eBioscience, Frankfurt, Germany) according to manufacturer’s recommendations. The LLD for the cytokine assay are the following: hIL-2: 16.4 pg/ml, hIL-4: 20.8 pg/ml, hIL-5: 1.6 pg/ml, hIL-6: 1.2 pg/ml, hIL-10: 1.9 pg/ml, hTNF-α: 3.2 pg/ml, hIFN-γ: 1.6 pg/ml, hIL-1β: 4.2 pg/ml, hIL-12p70: 1.5 pg/ml. To exclude cross-reactivity of the detection antibodies of the human FlowCytomix Th1/Th2 11plex kit with murine cytokines, the specificity of antibodies used to detect human cytokines was tested using murine cytokines mIL-1β, mIL-2, mIL-4, mIL-5, mIL-6 mIL-10, mIL-12p70, mTNF-α, and mIFN-γ in the same assay. Murine cytokine concentrations ranged from 69–50,000 pg/ml for mIL-1β and 27–20,000 pg/ml for all other murine cytokines (data not shown). Reagents used for this control were kindly provided by Lutz Kettner eBioscience, Frankfurt, Germany. Murine cytokines in mice sera were analyzed by ProcartaPlex Mouse Th1/Th2 Cytokine Panel (11 plex) plus ProcartaPlex Mouse IL-10 Simplex (eBioscience, Frankfurt, Germany) according to manufacturer’s recommendations.

### Human T cell purification and in vitro stimulation with coated OKT3

Human T cells were purified from hPBMCs by non-T cell depletion using the Pan T cell isolation kit II (Miltenyi Biotec, Bergisch Gladbach, Germany). Purity of human T cells usually exceeded 97%. All cells were cultured at 37°C with 5% CO_2_ in 24-well (1x10^6^ cells in 1 ml medium) flat-bottom tissue culture plates (Sarstedt, Nümbrecht, Germany) using X-VIVO 15 medium (Lonza, Basel, Switzerland). 24-well flat-bottom tissue culture plates (Sarstedt, Nümbrecht, Germany) were coated before with 5 μg/ml OKT3 (Janssen-Cilag, Neuss, Germany) in 200 μl PBS (Biochrom, Berlin, Germany) per well at 4°C for 24 h. Plates were washed twice with 400 μl PBS to remove unbound antibody prior to addition of human T cells in 1 ml X-VIVO 15 medium. Human T cells cultured on PBS-treated wells without antibody were used as negative control. CD3 expression on human T cells was measured by a flow cytometric analysis.

### Statistical analysis

Differences in cytokine levels were evaluated by means of a paired t-test for differences after-before. Percentages of hCD45^+^ cells and hCD3^+^ cells in different organs (spleen, PEC, LN, and thymus of humanized mice) were analyzed with a t-test, adjusted according Dunnett for multiple comparisons. For survival analysis, Kaplan-Meier curves were provided and comparisons between treatment groups were performed with a Logrank test, adjusted for multiple comparisons according Dunnett-Hsu. Correlation between cytokine amounts (or differences of cytokine levels) and hCD45^+^ cells were calculated with Spearman Rank Correlation Coefficient.

## Results

### Human T cells are the predominant cell population in hPBMC-reconstituted NOD-RAG1^-/-^Aβ^-/-^HLADQ^(tg+ or tg-)^IL-2Rγc^-/-^ mice

We selected NOD-RAG1^-/-^Aβ^-/-^HLADQ^(tg+ or tg-)^IL-2Rγc^-/-^ (NRG) mice and reconstituted them with human peripheral blood mononuclear cells (hPBMCs). In 88% of hPBMC-injected mice, human cells were detectable (meaning hCD45^+^ cells ≥15%; data not shown), and 8–16 days after reconstitution, those mice showed an average of 49% hCD45^+^ cells in peripheral blood (Fig 1B). Percentages refer to all cells (murine and human) in the peripheral blood of humanized mice since analyses using counting beads indicated that the absolute number of murine cells in the peripheral blood of NRG mice did not change with hPBMC and/or antibody injection and can thus serve as an internal reference (data not shown). The FACS gating strategy applied to all experiments throughout our study is given exemplarily in Fig 1A. In addition to peripheral blood, we detected hCD45^+^ cells in all organs analyzed (spleen 63%, peritoneal cavity 34%, lymph nodes 28%, and thymus 17%). As reported before, hCD3^+^ T cells were the predominant human cell type in humanized mice [16] with a CD4 to CD8 ratio comparable to hPBMC input (Fig 1C). Human CD19^+^ B cells were found mainly in spleen and lymph nodes (19% and 9%) whereas human CD11b^+^ myeloid cells were detectable predominantly in the peritoneal cavity (4%; Fig 1C). For all analyses throughout our study, humanized mice were used before onset of signs of severe graft versus host disease (GvHD). Collectively, hPBMC-reconstituted NRG mice fulfill all requirements for testing of T cell-specific mAbs in vivo.

### Lymphopenia and selective T cell loss from peripheral blood upon TGN1412 or OKT3 treatment of humanized mice

**Fig 2:**
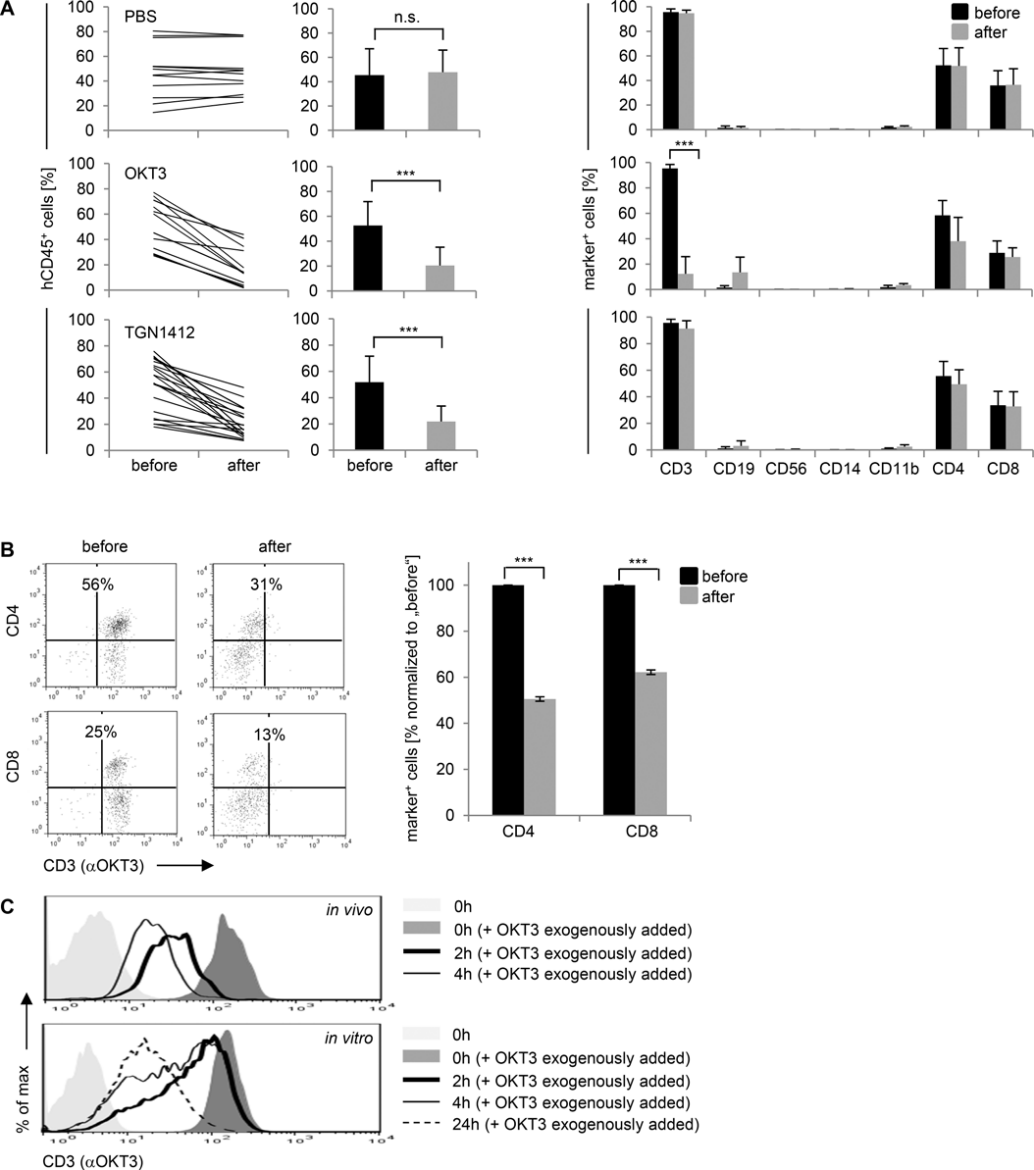
Loss of hCD45^+^ or hCD3^+^ cells from the peripheral blood upon TGN1412 or OKT3 treatment. Humanized mice were injected i.v. with 20 μg OKT3 or 20 μg TGN1412 per 10 grams body weight. **(A)** Before (black bars) and 2–6 hours post OKT3 (n = 11–12) or TGN1412 (n = 18–20) application (time point of sacrifice; gray bars), percentage and composition of hCD45^+^ cells in peripheral blood of reconstituted mice were analyzed by flow cytometric analysis. PBS-treated mice (n = 12) were used as control. Each line represents an individual mouse. *** p < 0.001; n.s., not significant (paired t-test for difference after-before). **(B)** Before and 4 hours post OKT3 application (n = 3) CD3 expression on hCD4^+^ or hCD8^+^ T cells in peripheral blood of humanized mice was investigated by flow cytometric analysis. Data from one individual animal, being representative for the indicated groups, are shown. The relative number of hCD4^+^ or hCD8^+^ T cells 4 hours after OKT3 application (gray bars) is given as percentages of total hCD4^+^ or hCD8^+^ T cells, normalized to “before”. *** p < 0.001 (paired t-test for difference after-before). **(C)** CD3 expression on human CD4^+^/CD8^+^ T cells in peripheral blood of humanized mice was investigated by flow cytometric analysis after OKT3 in vivo application (n = 3; upper panel). CD3 expression on CD4^+^/CD8^+^ T cells was investigated by flow cytometry before and after in vitro stimulation of purified human T cells with coated OKT3 (n = 3; lower panel). As control, primary human T cells were left unstained (light gray-shaded curve). Data from one individual animal or independent T cell donor, being representative for the indicated groups, are shown. Data shown in (A) are taken from 5–8 independent experiments. Data shown in (B) are representative for 2, in (C) for 3 independent experiments.

We injected humanized mice i.v. with TGN1412 or OKT3 using 20 μg mAb per 10 grams body weight. This dosage was selected in a way that both mAbs are comparable and both are close to concentrations applied to humans according to FDA guidelines for calculating the human equivalent dose based on body surface area [12]. Injection of humanized mice with either TGN1412 or OKT3 both resulted in lymphopenia 2–6 hours after antibody application: A loss of hCD45^+^ cells of about 60% from the peripheral blood was observed. Injection with PBS, low dose TGN1412 (1 μg/10 grams body weight), and Herceptin (20 μg/10 grams body weight) did not induce lymphopenia (Fig 2A and S1C and S1A Fig).

Analyzing human immune cell subsets in more detail revealed that upon TGN1412 injection, all cell subsets present in mice before treatment disappeared uniformly from peripheral blood since relative percentages of human CD3^+^, CD4^+^, CD8^+^, CD19^+^, and CD11b^+^ cells were comparable before and after TGN1412 treatment. In contrast, upon OKT3 injection of humanized mice, we observed reduced percentages of human CD3^+^ cells (95% before and 12% after treatment). Here, relative numbers of human CD19^+^ B cells increased, together indicating a selective loss of T cells. Human immune cell composition in peripheral blood of humanized mice remained unaltered upon PBS injection (Fig 2A).

Relative cell counts in Fig 2A were obtained from mice at the time point of sacrifice (2–6 hours post treatment). Because OKT3 injection of humans results in both T cell depletion and downmodulation of CD3 surface expression [17, 18], we analyzed CD3 expression on human CD4^+^ and CD8^+^ cells remaining in peripheral blood upon OKT3 treatment from additional mice that were sacrificed uniformly 4 hours post OKT3 treatment. As shown in Fig 2B, remaining CD4^+^ and CD8^+^ cells downregulated CD3 surface expression upon OKT3 treatment and both T cells subsets were indeed depleted from peripheral blood upon OKT3 injection.

Collectively, data shown here indicate that both mAbs applied to humanized mice induced effects such as lymphopenia or selective T cell ablation and downmodulation of CD3, which have been reported upon application of TGN1412 or OKT3 to humans, respectively [2, 17–19].

Upon OKT3 treatment of humans, T cell depletion and downmodulation of CD3 surface expression peaks only hours post injection [17–21]. Studying time kinetics indicated that already 2 hours post OKT3 injection of humanized mice, the vast majority of peripheral human CD4^+^/CD8^+^ T cells was negative for CD3 surface expression. 4 hours post injection, all CD4^+^/CD8^+^ T cells were negative for CD3 surface expression. In contrast, a comparable degree of CD3 downmodulation upon in vitro OKT3 stimulation of human T cells was achieved 24 hours after treatment (Fig 2C). Thus, one key feature of OKT3 treatment of humans, downmodulation of CD3 surface expression, can be achieved early in humanized mice as compared to an in vitro setting.

### OKT3-induced CD3 downmodulation on human T cells in solid organs of humanized mice

**Fig 3:**
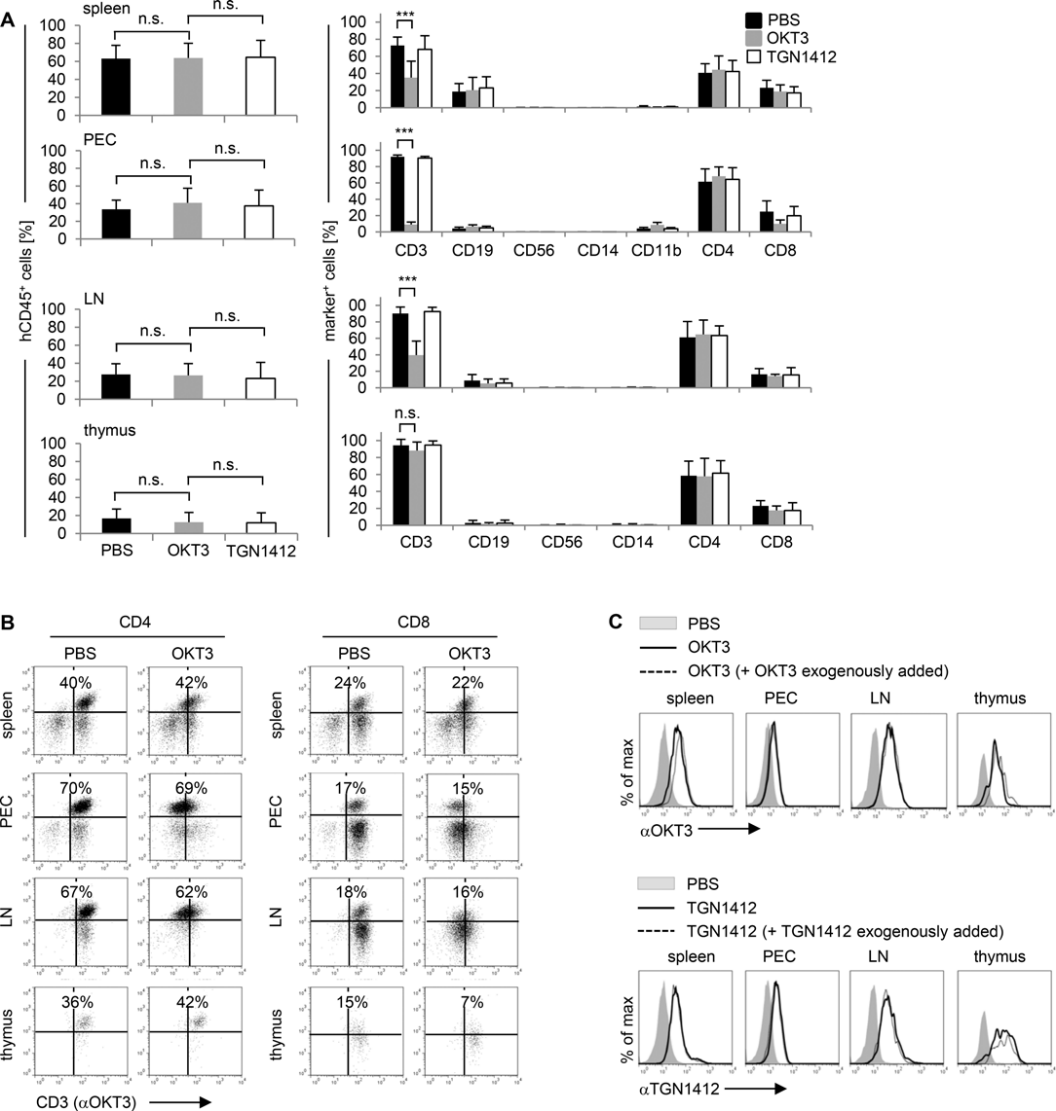
OKT3 application selectively downmodulates CD3 on T cells in humanized mice. Humanized mice were injected i.v. with 20 μg OKT3 or 20 μg TGN1412 per 10 grams body weight. **(A)** 2–6 hours (time point of sacrifice) post OKT3 (gray bars; n = 6–16) or TGN1412 (white bars; n = 6–16) application, percentage and composition of hCD45^+^ cells in spleen, PEC, LN, and thymus of humanized mice were analyzed by flow cytometry. PBS-treated mice (black bars; n = 6–16) were used as control. *** p < 0.001; n.s., not significant (t-test, for comparison of hCD45^+^ cells adjusted according Dunnett for multiple comparisons). **(B)** 4 hours post OKT3 (n = 3) application, CD3 expression on hCD4^+^ or hCD8^+^ T cells in spleen, PEC, LN, and thymus of humanized mice was analyzed by flow cytometry. PBS-treated mice (n = 3) were used as control to adjust quadrants for definition of positive and negative cells. Data from one individual animal, being representative for the indicated groups, are shown. **(C)** 4 hours post OKT3 (n = 3) or TGN1412 (n = 3) application, mAbs bound by their target CD3 or CD28 on human CD4^+^/CD8^+^ T cells in spleen, PEC, LN, and thymus of humanized mice were analyzed by flow cytometry (black curves). In vivo receptor occupancy of CD3 or CD28 was analyzed by exogenously adding OKT3 or TGN1412 to cells isolated from spleen, PEC, LN, and thymus of in vivo mAb-treated humanized mice (dotted curve). PBS-treated mice (n = 3) were used as control (gray-shaded curves). Data from one individual animal, being representative for the indicated groups, are shown. Data shown in (A) are taken from 2–6 independent experiments. Data shown in (B) and (C) are representative for 2 independent experiments.

Analyzing human cells in solid organs such as spleen, lymph nodes, and thymus and in the peritoneal cavity upon TGN1412 or OKT3 injection revealed that relative numbers of hCD45^+^ cells did not change upon mAb injection when compared to PBS control injection (Fig 3A). This indicated no cell loss, as observed in the peripheral blood upon TGN1412 and OKT3 injection. Even though we did not observe reduction of hCD45^+^ cells in solid organs upon mAb administration, we found reduction of CD3^+^ cells of up to 90% in spleen, peritoneal cavity, and lymph nodes upon OKT3 but not TGN1412 or PBS injection. No reduction in relative CD3^+^ cell counts was observed in the thymus (reduction of hCD8^+^ cells observed in individual mice (refer to Fig 3B) we attribute to very low total cell counts in thymus). In contrast to the peripheral blood (compare Fig 2A), relative numbers of all other human immune cell subsets remained unaltered indicating that CD3^+^ cells most probably were not depleted (Fig 3A). Data shown in Fig 3B confirmed that CD3 surface expression on human CD4^+^ and CD8^+^ T cells was downmodulated upon OKT3 treatment but, in contrast to the peripheral blood (compare Fig 2), cells were indeed not depleted from spleen, peritoneal cavity, lymph nodes, or thymus upon OKT3 treatment.

Because we observed downmodulation of CD3 surface expression on human T cells from spleen, lymph nodes, and the peritoneal cavity upon OKT3 injection but did not observe cell loss upon TGN1412 or OKT3 treatment and no effects of mAb injection in the thymus at all, we analyzed if and/or how efficient both mAbs had access to solid organs and the peritoneal cavity. Four hours post mAb injection, cells from spleen, the peritoneal cavity, lymph nodes, and thymus of humanized mice were harvested, and OKT3 or TGN1412 bound in vivo to human CD4^+^/CD8^+^ cells was detected by an anti-OKT3 or anti-TGN1412 antibody, respectively. Compared to PBS-injected humanized mice, we observed that OKT3 and TGN1412 indeed targeted CD4^+^/CD8^+^ cells in all organs analyzed (Fig 3C). To investigate in vivo receptor occupancy, we exogenously added OKT3 to cells harvested from OKT3-treated humanized mice and TGN1412 to cells harvested from TGN1412-treated humanized mice before staining with an anti-OKT3 or anti-TGN1412 antibody, respectively. Data given in Fig 3C (dotted lines) show that anti-OKT3 and anti-TGN1412 staining did not increase when mAbs were exogenously added indicating that in vivo receptor occupancy was almost complete. Thus, upon OKT3 or TGN1412 treatment of humanized mice, mAbs applied have access to solid organs and the peritoneal cavity, reach their target molecules CD3 or CD28 on human T cells, and receptor occupancy is close to 100%.

### TGN1412 and OKT3 treatment induces human cytokine release in humanized mice

**Fig 4:**
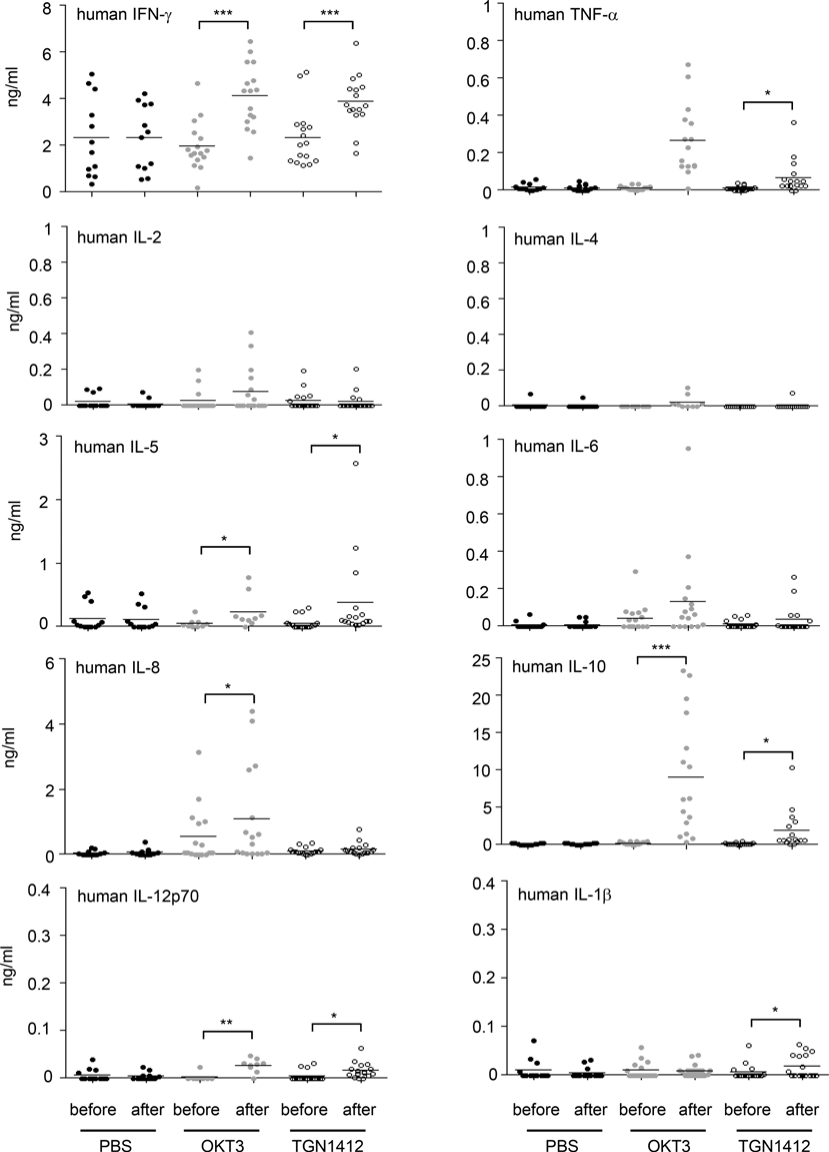
Release of human cytokines in humanized mice upon OKT3 and TGN1412 treatment. Humanized mice were injected i.v. with 20 μg OKT3 or 20 μg TGN1412 per 10 gram body weight. As control, humanized mice were injected with PBS. Before and 2–6 hours (time point of sacrifice) post OKT3 (n = 16) or TGN1412 (n = 16) application, blood was collected and investigated for human IFN-γ, TNF-α, IL-2, IL-4, IL-5, IL-6, IL-8, IL-10, IL-12p70, and IL-1β by human FlowCytomix Th1/Th2 11plex analysis. PBS-treated mice (n = 12) were used as control. *** p < 0.001; ** p < 0.01; * p < 0.05 (paired t-test for differences after-before). Data shown are taken from 4–6 independent experiments.

2–6 hours after TGN1412, OKT3, or PBS control injection, peripheral blood from treated animals was collected and analyzed for human cytokine expression. We found significant induction of human IFN-γ, TNF-α, IL-5, IL-8, IL-10, IL-12p70, and IL-1β upon injection of humanized mice with TGN1412 or OKT3, respectively. Of note, injection of PBS, low dose TGN1412, and Herceptin did not induce expression of any human cytokine analyzed (Fig 4 and S1D and S1B Fig). The human cytokine pattern induced differed slightly between TGN1412 and OKT3 treated mice with significant differences in TNF-α and IL-10 expression. With the exception of human IFN-γ, human cytokine expression was almost absent from peripheral blood of humanized mice before mAb injection (Fig 4). Data in S2 Fig exemplarily show expression of human IFN-γ before hPBMC injection, in humanized mice before treatment, and after OKT3 or TGN1412 injection for each individual mouse. These data indicate that cytokine induction could be observed in almost every individual mAb-injected animal (13 out of 15 animals treated with TGN1412 and 14 out of 15 OKT3-treated animals).

Detailed statistical analyses indicated that there was no correlation between the relative number of hCD45^+^ cells in peripheral blood before mAb treatment and the level of cytokine expression upon mAb administration (S3A Fig) suggesting that human cytokine expression was possible as soon as a certain threshold of human cell counts in humanized mice was reached. Interestingly, in TGN1412-treated animals we found an inverse correlation for IFN-γ secretion and lymphopenia. The more IFN-γ was detectable in an individual mouse, the less hCD45^+^ cells were found in peripheral blood, meaning the more pronounced lymphopenia was (S3B Fig).

### Humanized mice succumb to TGN1412 and OKT3 treatment

**Fig 5:**
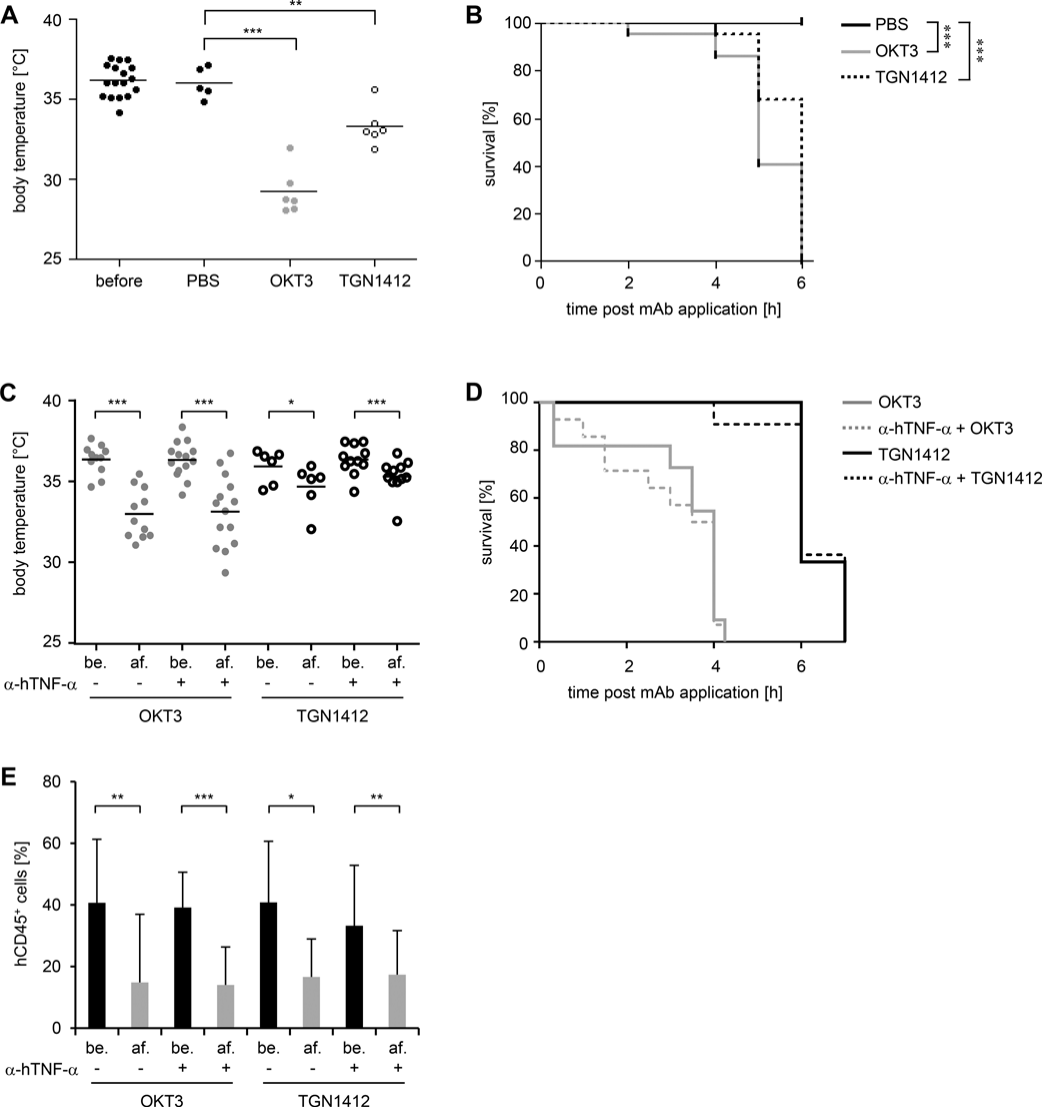
Humanized mice succumb to antibody application. Humanized mice were injected i.v. with 20 μg OKT3 or 20 μg TGN1412 per 10 grams body weight. 1 hour before, indicated humanized mice were treated with 10 μg anti-human TNF-α mAb per mouse (C-E). As control, humanized mice were injected with PBS (A-B). **(A)** Before and 2–6 hours (time point of sacrifice) post OKT3 (n = 6) or TGN1412 (n = 6) application, body temperature of mice was monitored. PBS-treated mice (n = 5) were used as control. ** p < 0.01; *** p < 0.001 (Dunnett-adjusted t-test). **(B)** 2–6 hours post OKT3 (n = 22) or TGN1412 (n = 22) application mice were sacrificed if severe signs of illness were detectable. PBS-treated mice (n = 18) were sacrificed 6 hours post treatment, the latest time point survived by mAb-treated mice. *** p < 0.001 (Dunnett-HSU-adjusted Logrank test). **(C)** Before (be.) and after (af.) OKT3 (n = 11) or TGN1412 (n = 6) application and 1 hour post anti-human TNF-α mAb (α-hTNF-α) application (before OKT3 or TGN1412 treatment) and 0.33–7 hours (time point of sacrifice) post OKT3 (n = 14) or TGN1412 (n = 11) application, body temperature of mice was monitored. * p < 0.05; *** p < 0.001 (paired t-test for difference after-before). **(D)** 0.33–7 hours post OKT3 (n = 11), anti-human TNF- α mAb + OKT3 (n = 14), TGN1412 (n = 6), or anti-human TNF-α mAb + TGN1412 (n = 11) application mice were sacrificed if severe signs of illness were detectable. **(E)** Before (be.) and after (af.) OKT3 (n = 11) or TGN1412 (n = 6) application and 1 hour post anti-human TNF-α mAb application (before OKT3 or TGN1412 treatment; black bars) and 0.33–7 hours post OKT3 (n = 14) or TGN1412 (n = 11) application (time point of sacrifice; gray bars), percentages of hCD45^+^ cells in peripheral blood of reconstituted mice were analyzed by flow cytometric analysis. * p < 0.05; ** p < 0.01; *** p < 0.001 (Wilcoxon signed rank test for difference after-before). Data shown in (A) are taken from 2, in (B) from 6–9, and in (C), (D), and (E) from 3 independent experiments.

Upon TGN1412 or OKT3 injection, humanized mice showed severe signs of illness such as limited mobility and ruffled fur (data not shown) and a massive drop of body temperature 2–6 hours after injection (Fig 5A). Finally, mice succumbed to TGN1412 and OKT3 application 2–6 hours after injection or had to be sacrificed, respectively (Fig 5B). As a control we used a 20-fold lower concentration of TGN1412 for treatment of humanized mice or the antibody Herceptin (20 μg per 10 grams body weight) for which no adverse events as those described for TGN1412 and OKT3 were reported upon administration to humans [22]. Here, treated mice did not show any signs of illness and did not succumb to treatment (data not shown). Hence, TGN1412 and OKT3 induce severe signs of illness in humanized mice and finally, animals succumb to treatment.

Because human TNF-α can function across species-specificity barriers [23, 24] we analyzed whether human TNF-α induced upon TGN1412 and OKT3 injection contributes to lethality of humanized mice after mAb injection. Therefore, humanized mice were treated with a human TNF-α neutralizing mAb (10 μg per mouse) 1 hour prior injection of TGN1412 or OKT3. 0.5–6 hours post OKT3 or TGN1412 application, mice showed a drop of body temperature (Fig 5C), severe signs of illness, and finally succumbed to TGN1412 or OKT3 injection (Fig 5D). In line with this, injection of humanized mice with either TGN1412 or OKT3 in anti-human TNF-α mAb pre-treated mice both resulted in lymphopenia 4–6 hours after antibody application (Fig 5E) as already described for TGN1412 or OKT3 injection alone (compare Fig 2A). Upon treatment with anti-human TNF-α mAb alone, the body temperature of those control-mice remained unaltered and animals did not show any signs of illness (data not shown). Thus, blocking human TNF-α does not influence lethality of humanized mice after TGN1412 or OKT3 treatment and hence, human TNF-α is most probably not the cause of the severe signs of illness observed upon mAb injection.

## Discussion

Briefly after the disastrous outcome of the first-in-human clinical trial with TGN1412, questions arose regarding preclinical testing of the mAb, since the macaque model, which was broadly considered as a kind of a gold-standard for preclinical safety evaluations, did not indicate any signs of toxicity or CRS when TGN1412 was administered [10]. In vitro studies using primary human T cells indicated that mainly CD4^+^CD45^+^CCR7^-^ effector memory T cells (T_EM_) respond to TGN1412 in terms of cytokine release [25]. Interestingly, macaque but not human CD4^+^ T cells lose CD28 expression during their differentiation into T_EM_ cells [25]. This might explain why the macaque model was not responsive to CD28-specific TGN1412 and hence, was not predictive as a pre-clinical model for the severe adverse events induced upon TGN1412 administration to humans. Also, laboratory rodents are not useful as a pre-clinical model for testing reagents such as TGN1412 relying on T_EM_ cells. Those animals are housed under clean, specific pathogen free (SPF) conditions, not exposed to infectious agents and hence, they do not develop T_EM_ cells [5]. We used a humanized mouse model in order to evaluate its predictive value for mAb preclinical testing. Using a humanized mouse model in which animals are reconstitution with CD34^+^ stem cells we consider as not suitable. These animals will not develop T_EM_ since they have to be housed under SPF conditions. Therefore, we selected NRG mice reconstituted with hPBMCs in order to have at least low numbers of transferred T_EM_ present in the animals.

Using humanized mice to analyze effects of therapeutic mAbs became more successful in the last years [26, 27]. For example, Brady and colleagues observed adverse effects upon injection of OKT3, Campath-1H or the polyclonal Ab preparation anti-thymocyte globulin in a humanized SCID model [3]. In line with this, analyzing serum samples upon TGN1412 or OKT3 injection of humanized mice, we detected expression of a broad range of human cytokines already 2–6 h after mAb application (Fig 4). Compared to data published upon the first-in-human clinical trial with TGN1412 [2], we found all cytokines analyzed to be expressed in humanized mice with the exception of IL-2 and IL-4. Nevertheless, absolute levels of cytokines were lower in humanized mice when compared to the volunteers, which can be expected due to far less cells in mice than in adult humans. Data obtained from a clinical trial with OKT3 indicated serum TNF-α levels between 144 and 2283 pg/ml and rather low levels of IFN-γ (169 pg/ml) [28, 29]. However, IFN-γ levels observed in humanized mice in our study are more than an order of magnitude higher (Fig 4 and [30]).

Using 5.11A1 (a precursor to TGN1412; a mouse IgG1 mAb directed to human CD28) in humanized mice, human cytokine release has not been investigated or reported, respectively [31]. Treatment of rats with the mAb JJ316 (a rat CD28-specific mAb homologous to TGN1412) induced rat TNF-α (0.4 ng/ml) and rat IFN-γ (0.07 ng/ml) expression within 24 hours after injection [32]. Compared to our data, induction of TNF-α was higher (Fig 4) whereas IFN- γ levels were lower (Fig 4) in JJ316-treated rats than in TGN1412-treated humanized mice. Of note and in contrast to our study, rats did not succumb to JJ316 treatment [32].

Malcolm et al. studied effects of certain CD3-specific antibody fragments including OKT3 in a humanized mouse model of CB17-SCID mice injected with hPBMCs along with tetanus toxoid. In addition to expression of human IFN-γ, IL-10, and TNF-α, some IL-2 release was detected in that particular study 2–4 h post OKT3 injection. However, human T cell depletion in blood, spleen, and peritoneum was observed 5 days post OKT3 treatment. In that study, mice did not succumb to OKT3 treatment which might be related to a rather low dose of 5 μg OKT3 per mouse which was administered [30]. In our study, we followed the FDA guideline for calculation of the human equivalent dose [12] to determine OKT3 and TGN1412 dosages applied to humanized mice. By administering a 20-fold lower dose of TGN1412, no effects such as signs of illness, lymphopenia or human cytokine release could be detected (S1C and S1D Fig). Only 2–6 hours post application of TGN1412 or OKT3 mice showed severe signs of illness, a drop in body temperature, and finally succumbed to mAb application (Fig 5). Hence, using this model system, no previous knowledge on adverse events to be expected and molecular mechanisms involved is needed, in order to predict severe adverse events induced upon mAb injection.

Since human TNF-α is cross-reactive on the murine system [23, 24] we treated humanized mice with a human TNF-α neutralizing mAb prior to injection of TGN1412 or OKT3 to analyze the contribution of human TNF-α to the severe signs of illness induced upon TGN1412 and OKT3 administration. However, anti-human TNF-α mAb pre-treated mice showed signs of illness comparable to mice injected with TGN1412 or OKT3 alone and finally, also those mice succumbed to mAb treatment (Fig 5D). Of note, human IFN-γ, also released upon mAb treatment, is only active on human cells, but not on cells of other mammalian species such as the mouse and rat [33]. Thus, it is not clear yet, whereof mice finally die upon TGN1412 and OKT3 treatment in our study. Both a mediator released by human or by murine cells could be involved in the rapid lethality of mice after antibody injection. The induction of murine cytokines upon TGN1412 and OKT3 treatment is likely not to be responsible for lethality. Analyzing murine IFN-γ, TNF-α, GM-CSF, IL-1β, IL-2, IL-4, IL-5, IL-6, IL-10, IL-12p70, IL-13, and IL-18, the only murine cytokine we found to be induced in relevant amounts was murine IL-6. However, induction was observed only upon OKT3 but not upon TGN1412 injection. Murine IL-18 was also present in high amounts in humanized mice, but levels were unaffected by PBS, OKT3, or TGN1412 injection (S4). In line with this, we did not observe differences in the number of murine CD45^+^ cells upon mAb injection (data not shown) which otherwise could be a hint for effects of murine cytokines on the murine cell system.

In a recent study by Tabares and colleagues, 1000- to 15-fold lower doses of TGN1412 (now renamed TAB08) were applied to healthy volunteers and did not lead to the release of pro-inflammatory cytokines TNF-α, IFN-γ, and IL-2. Interestingly, at 5 μg/kg, a dose 20-fold below the one applied in the trial of TGN1412 in 2006, an IL-10 response of about 7 pg/ml peaked 12 hours after infusion [34]. In line with this, in humanized mice treated with a 20-fold lower dose TGN1412 we did not observe any signs of illness (data not shown). Moreover, 20-fold lower dose TGN1412 injection did neither induce expression of any human cytokines analyzed (S1D Fig). However, it cannot be excluded that levels of about 7 pg/ml IL-10 are not measurable due to the detection limit of our assay system.

We and others showed that TGN1412 treatment of peripheral human T cells in vitro does not induce T cell activation, proliferation, or cytokine release. A second stimulatory signal such as TGN1412 crosslinking [35–38], ICOS:LICOS interaction between TGN1412-treated T cells and endothelial cells [38], or a weak or “tonic” TCR signaling [39] is needed to fully induce TGN1412-mediated T cell responses. Likewise, Smith et al. showed that OKT3 preferentially induced cytokine release from activated but not from naive T cells [40]. We analyzed human T cell activation in our humanized mouse model. Already before mAb treatment, human T cells showed a highly activated phenotype (data not shown). This pre-activated status of human T cells is in line with elevated serum levels of human IFN-γ detectable already before mAb treatment (Fig 4, S2 Fig). These elevated basal levels of serum IFN-γ and the activated phenotype of human T cells after hPBMC transfer but before mAb treatment might be related to the onset of GvHD, which is reported for humanized mouse models [41, 42]. This xenoreactive activation of T cells would of course not be found in human recipients. Thus, pre-activation of human T cells in our model of humanized mice might amplify effects mediated by TGN1412 and OKT3 such as lymphopenia and human cytokine release. The fast occurrence of a GvHD could be prevented using humanized mice with a human hematopoietic stem cell-derived immune system. However, testing mAb TGN1412 in this system most probably will fail due to the lack of T_EM_ as discussed above.

In our study, humanized mice showed loss of hCD45^+^ cells from the peripheral blood 2–6 hours post TGN1412 treatment. Humanized mice have been employed previously to analyze effects of the anti-CD28 superagonistic mAb 5.11A1 [31]. In this study, immunodeficient BALB/c Rag2^-/-^γ c^-/-^ mice were reconstituted with human CD34^+^ fetal liver stem cells. Here, depletion of circulating human CD28^+^ T cells could be detected between day 3 and day 60 after antibody application. This rather late depletion of T cells upon 5.11A1 application might pinpoint towards antibody-dependent cell-mediated depletion or complement-mediated depletion rather than lymphopenia; particularly, since 5.11A1 is an IgG1 whereas TGN1412 is an IgG4 mAb. Interestingly, remaining cells in all organs analyzed were negative for annexin-5 suggesting that activation-induced cell death might not account for cell loss observed in our experimental setting (data not shown).

For OKT3 administration it has been reported that T cell depletion from the peripheral blood can be observed already 10–20 minutes after injection of patients [17, 21]. Moreover, 48 hours after terminating OKT3 administration, T cells are again detectable in peripheral blood. Whether in our setting, human CD45^+^ cells are depleted upon OKT3 and TGN1412 treatment or whether cells just disappear from peripheral blood and reenter after discontinuing treatment cannot be investigated since mice have to be sacrificed due to severe signs of illness at too early time points after mAb injection (Fig 5).

Analyzing human cells in solid organs and the peritoneal cavity of humanized mice, we did not observe any changes in hCD45^+^ cell counts. This might indicate that cells lost from the peripheral blood do not enter solid organs. However, in the human body, circulating blood contains 2–5% of all lymphocytes [43, 44]. That we detect loss of human CD45^+^ cells from the peripheral blood but cannot detect any accumulation of lymphocytes in organs after mAb treatment may thus be related to the small number of T cells in the blood of humanized mice as well.

Of note, we did observe CD3 downmodulation on T cells from spleen, lymph nodes, and the peritoneal cavity upon OKT3 injection (Fig 3B). We suggest that the mAbs might reach solid organs delayed when compared to the peripheral blood. Using the mAb JJ316 in rats, Müller et al. observed a drop of CD4^+^ T cell counts in peripheral blood 4 hours after administration and an increase of T cells in lymph nodes and spleen 72 hours post JJ316 treatment, a time point humanized mice already succumbed to mAb treatment in our study. Of note, 10 hours post JJ316 injection, no increase in CD4^+^ T cell counts in secondary lymphatic organs was detected [32].

Collectively, we described a humanized mouse model for preclinical testing of therapeutic mAbs in which a number of effects and severe adverse events induced upon mAb treatment of patients or volunteers could be mimicked. However, we chose a mouse model in which T cells are the main human cell type present (Fig 1C) and used two T cell-specific mAbs for analyses. It will be a matter of future investigations to establish models suitable for analyzing mAbs which have other cells than T cells as target population.

## Supporting Information

S1 Fig Injection with low TGN1412 or Herceptin did not induce lymphopenia or cytokine release. Humanized mice were injected i.v. with 20 μg Herceptin or 1 μg TGN1412 (low) per 10 grams body weight. Before (black bars) and 6 hours post Herceptin ((A); n = 5) or low TGN1412 ((C); n = 7) application (gray bars), percentages of hCD45^+^ cells in peripheral blood of reconstituted mice were studied by flow cytometric analysis. Before and 6 hours post Herceptin ((B); n = 4) or low TGN1412 ((D); n = 7) application, blood was collected and investigated for human IFN-γ, TNF-α, IL-10, and IL-6 by human FlowCytomix Th1/Th2 11plex analysis. Data shown in (A) and (B) are taken from 2 independent experiments, in (C) and (D) from 3 independent experiments.
(PPTX)

S2 Fig Serum levels of human IFN-γ in individual mice. Humanized mice were injected i.v. with 20 μg OKT3 or 20 μg TGN1412 per 10 gram body weight. Before reconstitution, before mAb application, and 2–6 hours (time point of sacrifice) post OKT3 (n = 16) or TGN1412 (n = 16) application blood was collected and analyzed for human IFN-g by human FlowCytomix Th1/Th2 11plex analysis. Each line represents an individual mouse.
(PPTX)

S3 Fig No correlation between the number of hCD45^+^ cells and the level of cytokine expression. **(A)** Data obtained by analyzing hCD45^+^ cells in peripheral blood and cytokine expression upon mAb administration were statistically analyzed (Spearman Rank Correlation Coefficient) indicating no correlation between hCD45^+^ cell counts in peripheral blood before mAb treatment and the level of IFN-g or TNF-a expression upon TGN1412 or OKT3 administration. **(B)** Data obtained analyzing cytokine expression and lymphopenia upon mAb administration were statistically analyzed (Spearman Rank Correlation Coefficient) indicating an inverse correlation between IFN-γ secretion and hCD45^+^ cell counts in peripheral blood after TGN1412 (but not OKT3) treatment.
(PPTX)
