# Supplementary material for: TGN1412 Induces Lymphopenia and Human Cytokine Release in a Humanized Mouse Model
Source: PLoS One. 2016 Mar 9;11(3):e0149093. doi: 10.1371/journal.pone.0149093 (PMC4784892; doi:10.1371/journal.pone.0149093)
Supplement: S1 Fig — Humanized mice were injected i.v. with 20 μg Herceptin or 1 μg TGN1412 (low) per 10 grams body weight. Before (black bars) and 6 hours post Herceptin ((A); n = 5) or low TGN1412 ((C); n = 7) application (gray bars), percentages of hCD45+ cells in peripheral blood of reconstituted mice were studied by flow cytometric analysis. Before and 6 hours post Herceptin ((B); n = 4) or low TGN1412 ((D); n = 7) application, blood was collected and investigated for human IFN-γ, TNF-α, IL-10, and IL-6 by human FlowCytomix Th1/Th2 11plex analysis. Data shown in (A) and (B) are taken from 2 independent experiments, in (C) and (D) from 3 independent experiments. (PPTX) [file pone.0149093.s001.pptx]

## Slide 1
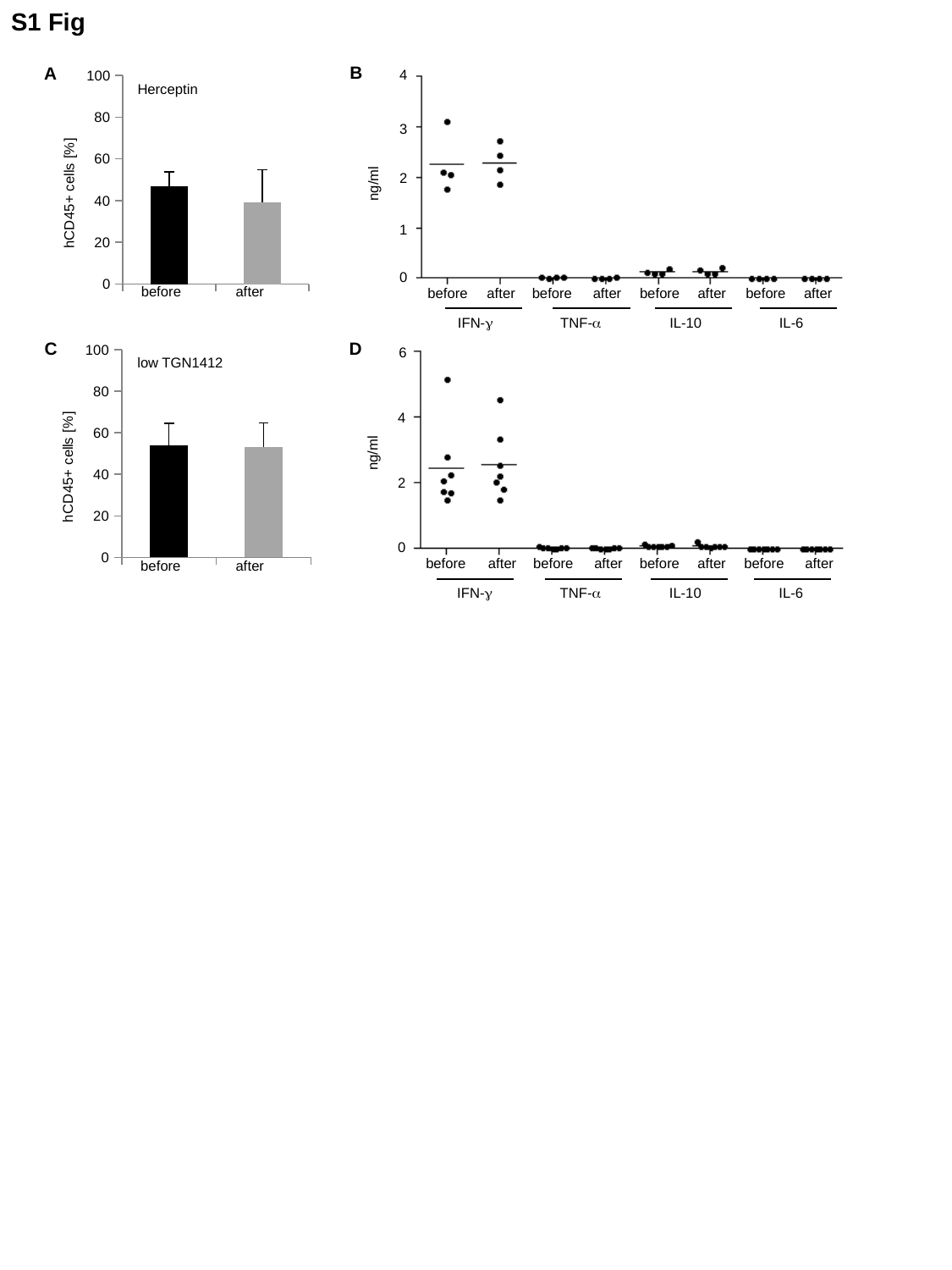

S1 Fig
B
A
4
### Chart
| Category | |
|---|---|
| before | 46.940000000000005 |
| after | 39.22 |
Herceptin
3
2
ng/ml
1
0
before
after
before
after
before
after
before
after
before
after
IFN-g
TNF-a
IL-10
IL-6
C
D
### Chart
| Category | |
|---|---|
| before | 53.91428571428572 |
| after | 53.042857142857144 |6
low TGN1412
4
ng/ml
2
0
before
after
before
after
before
after
before
after
before
after
IFN-g
TNF-a
IL-10
IL-6
