# Supplementary material for: TGN1412 Induces Lymphopenia and Human Cytokine Release in a Humanized Mouse Model
Source: PLoS One. 2016 Mar 9;11(3):e0149093. doi: 10.1371/journal.pone.0149093 (PMC4784892; doi:10.1371/journal.pone.0149093)
Supplement: S2 Fig — Humanized mice were injected i.v. with 20 μg OKT3 or 20 μg TGN1412 per 10 gram body weight. Before reconstitution, before mAb application, and 2–6 hours (time point of sacrifice) post OKT3 (n = 16) or TGN1412 (n = 16) application blood was collected and analyzed for human IFN-g by human FlowCytomix Th1/Th2 11plex analysis. Each line represents an individual mouse. (PPTX) [file pone.0149093.s002.pptx]

## Slide 1
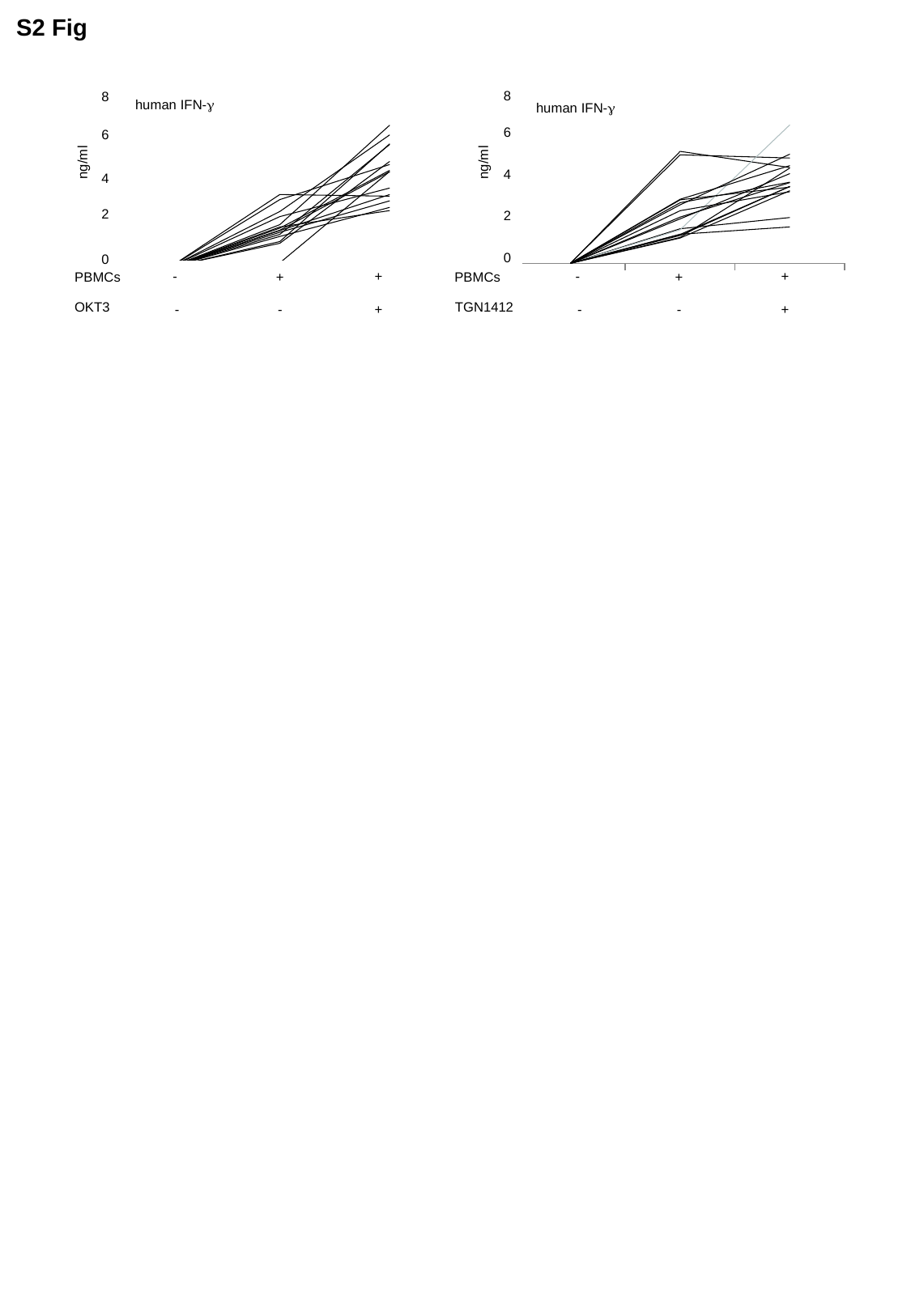

S2 Fig
### Chart
| Category | 6 Exp 75 / 3#1 | 6 Exp 75 / 4#1 | 6 Exp 75 / 2#1 | 6 Exp 75 / 4#3 | 6 Exp 81 / 2#1 | 6 Exp 82 / 1#3 | 6 Exp 84 / 1#1 | 6 Exp 87 / 5#1 | 6 Exp 87 / 5#2 | 5 Exp 73 / 1#1 | 5 Exp 73 / 9#1 | 5 Exp 73 / 1#2 | 5 Exp 73 / 3#1 | 5 Exp 73 / 7#1 | 5 Exp 73 / 8#1 | 4 Exp 82 / 2#1 |
|---|---|---|---|---|---|---|---|---|---|---|---|---|---|---|---|---|
| vor PBMCs | 1.37 | 0.03 | 0.0 | 0.0 | 0.0 | 0.0 | 0.0 | 0.0 | 0.0 | 0.0 | 0.0 | 0.0 | 0.0 | 0.0 | 0.0 | 0.0 |
| 0 | 1589.28 | 5001.36 | 2790.55 | 5159.25 | 1543.09 | 2922.58 | 2949.83 | 2694.03 | 2046.04 | 1328.46 | 2126.66 | 1280.56 | 1162.93 | 2423.39 | 1189.9 | 1336.83 |
| nach hTGN1412 | 2106.98 | 4853.44 | 3736.34 | 4413.89 | 6383.96 | 3511.68 | 4503.26 | 5030.04 | 4134.89 | 3542.6 | 3718.21 | 3542.6 | 3343.99 | 3296.13 | 4392.03 | 1672.6 |
### Chart
| Category | 6 # 61 K1#1 | 6 # 61 K3#1 | 6 # 61 K3#2 | 6 # 61 K3#3 | 6 # 61 K4#1 | 6 # 61 K4#2 | 6 # 61 K4#3 | 6 Exp 75 / 6#2 | 6 Exp 75 / 7#1 | 5 Exp 82 / 2#2 | 5 Exp 82 / 2#3 | 5 Exp 84 / 2#1 | 5 Exp 84 / 2#2 | 4 Exp 75 / 6#1 | 2 Exp 81 / 5#1 |
|---|---|---|---|---|---|---|---|---|---|---|---|---|---|---|---|
| vor PBMCs | 0.0 | 0.0 | 0.0 | 0.0 | 0.0 | 0.0 | 0.0 | 9.0 | 12.0 | 0.0 | 0.0 | 0.0 | 0.0 | 0.0 | 0.0 |
| 0 | 2294.6 | 1394.67 | 1583.36 | 1824.1 | 1661.49 | 1676.12 | 3298.76 | 194.0 | 1085.0 | 1162.57 | 2527.74 | 1772.82 | 1930.22 | 3068.0 | 1488.76 |
| nach OKT3 | 3595.14 | 2713.24 | 3010.9 | 2568.29 | 3298.62 | 4336.39 | 3221.58 | 4348.0 | 4804.0 | 5602.7 | 6007.79 | 4397.27 | 6449.09 | 4662.0 | 5575.1 |8
8
human IFN-g
human IFN-g
6
6
ng/ml
ng/ml
4
4
2
2
0
0
-
+
-
+
+
+
PBMCs
PBMCs
OKT3
TGN1412
-
+
-
+
-
-
