# Supplementary material for: TGN1412 Induces Lymphopenia and Human Cytokine Release in a Humanized Mouse Model
Source: PLoS One. 2016 Mar 9;11(3):e0149093. doi: 10.1371/journal.pone.0149093 (PMC4784892; doi:10.1371/journal.pone.0149093)
Supplement: S3 Fig — (A) Data obtained by analyzing hCD45+ cells in peripheral blood and cytokine expression upon mAb administration were statistically analyzed (Spearman Rank Correlation Coefficient) indicating no correlation between hCD45+ cell counts in peripheral blood before mAb treatment and the level of IFN-g or TNF-a expression upon TGN1412 or OKT3 administration. (B) Data obtained analyzing cytokine expression and lymphopenia upon mAb administration were statistically analyzed (Spearman Rank Correlation Coefficient) indicating an inverse correlation between IFN-γ secretion and hCD45+ cell counts in peripheral blood after TGN1412 (but not OKT3) treatment. (PPTX) [file pone.0149093.s003.pptx]

## Slide 1
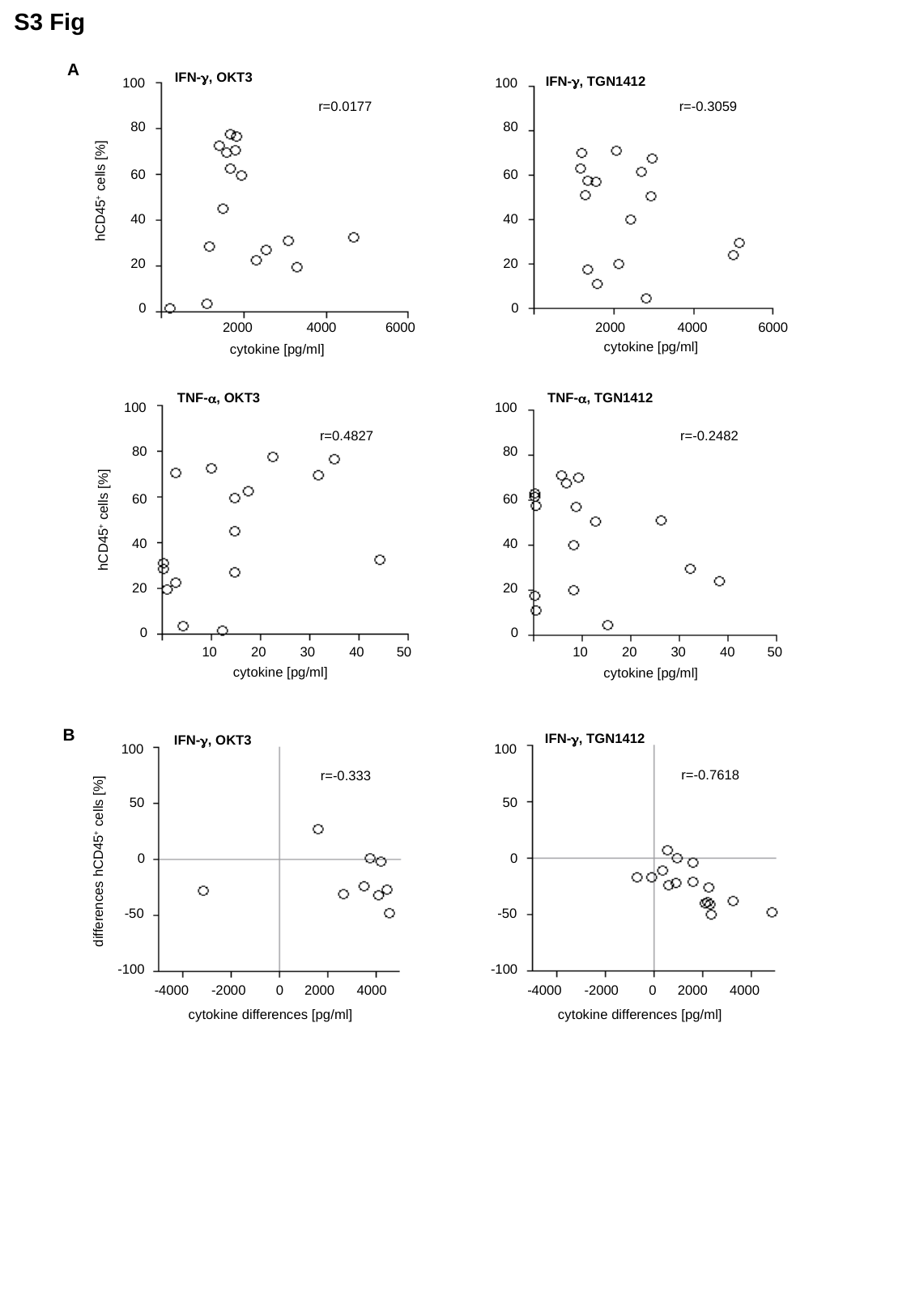

S3 Fig
A
IFN-g, OKT3
100
r=0.0177
80
60
hCD45+ cells [%]
40
20
0
2000
4000
6000
cytokine [pg/ml]
IFN-g, TGN1412
100
r=-0.3059
80
60
40
20
0
2000
4000
6000
cytokine [pg/ml]
TNF-a, OKT3
100
r=0.4827
80
60
hCD45+ cells [%]
40
20
0
10
20
30
40
50
cytokine [pg/ml]
TNF-a, TGN1412
100
r=-0.2482
80
60
40
20
0
10
20
30
40
50
cytokine [pg/ml]
B
IFN-g, OKT3
100
r=-0.333
50
0
differences hCD45+ cells [%]
-50
-100
-4000
-2000
0
2000
4000
cytokine differences [pg/ml]
IFN-g, TGN1412
100
r=-0.7618
50
0
-50
-100
-4000
-2000
0
2000
4000
cytokine differences [pg/ml]
